# Project-based learning as a catalyst for 21st-Century skills and student engagement in the math classroom

**DOI:** 10.1016/j.heliyon.2024.e39988

**Published:** 2024-11-15

**Authors:** Nadia Rehman, Xiao Huang, Amir Mahmood, Mohammed A.M. AlGerafi, Saima Javed

**Affiliations:** College of Education, Zhejiang Normal University, Jinhua, Zhejiang, China

**Keywords:** Project-based learning, Collaborative learning, Problem-solving skills, Critical thinking skills, Structural equation modeling (SEM)

## Abstract

Along with traditional academic knowledge, 21st-century skills are crucial for equipping students with a competitive future. Project-based learning (PBL) cultivates these competencies among learners as an instructional approach. This research aimed to construct and analyze a PBL framework designed to weave 21st-century skills into high school education. Data collection involved a survey questionnaire based on an extensive literature review administered to students from ten government-run girls' high schools, where mathematics was taught using the PBL approach. Employing Confirmatory Factor Analysis (CFA) and Structural Equation Modeling (SEM) via the Analysis of Moment Structures (AMOS) software, the study meticulously calculated chi-square values, modification indices, and standardized estimates to validate the framework's effectiveness. The results showed a significant enhancement in collaborative learning, problem-solving, critical thinking, and positive attitudes toward mathematics among students, mediated by the PBL approach. The Heterotrait-Monotrait Ratio (HTMT) values were below the threshold of 1, confirming discriminant validity and ensuring that each construct was unique. The observed substantial correlations between PBL and educational outcomes, ranging from moderate to significant, attest to these variables' interconnectedness and mutual reinforcement. Overall, the structural model and subsequent analyses underscore PBL's pivotal role in promoting active learning and student engagement. This approach advocates its adoption as a forward-thinking educational strategy.

## Highlights

• Project-Based Learning Enhances Collaborative Skills.
• PBL Strengthened Problem-Solving Abilities.
• PBL Amplified Critical Thinking Skills.
• PBL develops a Positive Shift in Math Attitude.
• PBL Increased Student Engagement.

## 1 Introduction

Education is a dynamic field in which innovative approaches are constantly sought to engage students and foster learning [1]. One such approach is PBL [2]. PBL emphasizes active, hands-on learning experiences that empower students to tackle real-world problems, collaborate with peers, think critically, and develop 21st-century skills [3]. This approach diverges from conventional teaching by engaging learners in real-world situations, prioritizing experiential learning, teamwork, critical analysis, and creating a dynamic educational environment [4].

As educators strive to enhance student engagement (SE) and cultivate a deeper understanding of the subject matter, investigating the effectiveness of PBL becomes crucial [5]. Studies have shown that PBL helps students develop essential skills such as collaborative skills (CL), problem-solving (PSS), and critical thinking (CTK), making it a powerful approach for various subjects, including mathematics [6]. In mathematics, PBL allows learners to delve into mathematical principles in tangible ways by leveraging them in real-world scenarios, thus closing the gap between abstract knowledge and practical application [7].

One of the significant benefits of PBL in mathematics education is its potential to enhance comprehension of mathematical principles through practical projects that uncover logical truths within their context [8]. This active engagement encourages a well-rounded understanding of mathematical ideas as students see their practicality and significance in real-world situations. Additionally, PBL sharpens critical thinking abilities as students confront real-life challenges, prompting them to dissect, assess, and employ mathematical approaches and logical reasoning [9]. They become adept at pattern recognition, establishing connections, and forming logical arguments to solve complex problems.

Moreover, PBL encourages collaboration, a critical attribute significantly enhancing students' mathematical understanding. Teamwork and collective effort are often crucial when tackling real-life issues, providing a conducive environment for collaboration, conversation, and collective goal achievement [10]. Participating in collaborative projects exposes students to various perspectives, enabling them to compromise on ideas and participate in constructive debates. This approach amplifies mathematical comprehension and cultivates crucial interpersonal skills imperative in today's workforce [4].

Finally, PBL can enhance students' attitudes toward mathematics. Conventional teaching methods often fall short of engaging students or demonstrating the subject's practical importance, whereas PBL motivates students by presenting real-world challenges that require mathematical reasoning [11]. When students perceive the direct application of mathematical concepts in real-life scenarios, they are more likely to engage with the subject and cultivate a positive outlook toward mathematics [12].

The transformative power of PBL in mathematics education is undeniable. It actively engages students, enhances their understanding of mathematical concepts, fosters CTK promotes effective CL, and ultimately increases their engagement in learning [13]. This teaching strategy breathes life into mathematics by anchoring it in the real-world context through student-centric projects, making the subject matter relevant and meaningful to students' everyday experiences [14]. As we continue shaping robust and enriching educational experiences, PBL emerges as a promising approach, empowering students to cultivate their math skills, gain confidence, and find joy in learning [15].

### 1.1 Research gap and problem statement

Despite the growing emphasis on PBL in educational research, empirical studies on its specific impact on developing 21st-century skills, such as PSS, CTK, and CL, remain limited, particularly within mathematics education. While many studies have explored the general effectiveness of PBL in classroom settings, few have rigorously examined its role in cultivating these essential skills in public school systems, particularly in under-researched contexts such as low-resource environments.

Furthermore, SE is a critical factor in improving learning outcomes and has been underexplored in relation to PBL implementation in mathematics curricula. A clear need exists to understand how PBL shapes student attitudes, motivation, and active participation, particularly where students have limited access to innovative teaching methodologies.

This study addresses these gaps by developing a comprehensive model that evaluates not only the impact of PBL on acquiring 21st-century skills but also how these skills contribute to enhancing SE in mathematics learning. Focusing on girls' schools in Islamabad, this research also responds to the scarcity of studies examining gender-specific educational interventions. The findings aim to provide valuable insights into tailoring educational interventions that improve outcomes for female students.

**Novelty of the Study:** This study provides a novel contribution to educational research by developing and empirically validating a comprehensive model that examines how PBL influences 21st-century skills and SE in mathematics education. While previous research has explored PBL’s effectiveness in general educational settings, few studies have rigorously evaluated its specific impact on these key skills in **government-run schools in Pakistan**, particularly in **girls’ schools**. This focus on a gender-specific context offers new insights that have received limited attention in PBL research. Furthermore, using CFA and SEM strengthens the study's novelty by ensuring a robust methodological approach and a deep understanding of the relationship between PBL and student outcomes.

**Purpose of the Study:** This study aims to develop and validate a comprehensive model that analyzes the influence of PBL on students’ 21st-century skills and how these skills affect SE in mathematics learning. Data were collected from 600 students in ten government-run high schools in Islamabad, where PBL was integrated into the mathematics curriculum over six weeks. Using CFA and SEM, the study rigorously evaluates the model’s validity and reliability, offering valuable insights into the effectiveness of PBL in promoting both skill development and SE.

This research investigates the intricate relationship between PBL and SE in mathematics, providing critical insights for developing effective teaching methods. The study contributes to the existing body of knowledge by illuminating how PBL fosters SE, with implications for curriculum design and instructional strategies. The findings hold the potential to inform educational practices, empowering educators to craft instructional strategies that enhance student motivation, learning, and overall engagement.

## 2 Literature review and hypothesis development

Since the 1990s, PBL has gained popularity in various academic fields related to teaching [16]. Stemming from John Dewey's learning-by-doing concept, it was initially used in medicine and engineering during the 1970s [17]. PBL fosters students in design, problem-solving, and decision-making processes [18], with teachers facilitating learning rather than directly teaching content [19]. PBL learning combines knowledge acquisition with professional development, using real-world problems as the foundation for learning. It fosters active, interactive, and collaborative learning [3], improving critical and analytical thinking skills [20]. Studies show PBL's superiority over traditional methods as it teaches students to find information, solve problems, make decisions, and work in teams [21,22]. Furthermore, PBL facilitates understanding previous concepts and acquiring updated knowledge [23].

PBL is an innovative blend of practical, hands-on approaches that entail teamwork, collaboration, and an intellectual, mind-on strategy that promotes authentic problem-solving and critical thinking. This unique blend has proven effective in helping students delve deeper into mathematical concepts and understand them better, as Chen and Yang suggested [24]. This approach also helps students remember their lessons for longer durations and enhances their ability to apply knowledge in real-life situations [25]. Advocates of PBL believe that students studying in institutions implementing PBL pedagogy demonstrate increased motivation to learn the information required for their projects [26]. As an illustration, Wolpert-Gawro indicated that PBL inspired students to tackle challenges head-on instead of shying away from them, especially in math education [27]. This type of learning allows students to absorb subject matter effectively, retain it for extended periods, excel in examinations, and apply their skills to various scenarios. Arguably, the most impactful outcome of a superior educational method is the long-term enhancement of students' learning process [28].

The social constructivist approach aligns with PBL, emphasizing student agency, group work, and guidance [29]. PBL encourages active involvement in real-world projects, developing transferable skills and interpersonal learning [30,31]. This transformative education results in long-term knowledge retention and commitment to a democratic society [32].

Gardner's multiple intelligence theory complements PBL, recognizing eight types of intelligence in students [33]. PBL accommodates various learning preferences [34]. Kolb's Experiential Learning Theory (ELT) is a PBL foundation highlighting children's innate curiosity and engagement with the world [35]. ELT emphasizes a meaningful learning environment and real-world connections [25]. Students experience a sense of belonging when working toward common goals [35]. PBL fosters meaningful learning by building on existing knowledge and participating in globally significant projects [36].

PBL is not a novel pedagogical approach but is deeply rooted in educational theory and practice. The significance of PBL in fostering 21st-century skills has been the subject of rigorous inquiry in previous studies, which provided a rich context for current research. Prior investigations have explored the multifaceted dimensions of PBL, examining its potential to cultivate collaboration, problem-solving abilities, critical thinking, and positive attitudes toward subjects such as mathematics. The empirical literature on PBL has extensively documented its efficacy in promoting 21st-century competencies among students. Recent studies have employed diverse methodologies to probe the extent to which PBL impacts educational outcomes. For instance, Rehman et al. utilized an experimental design to assess collaborative skill development in students engaged in PBL activities [3]. Their findings indicated a significant improvement in students' ability to work in teams over time. This finding suggested that PBL has lasting effects on skill acquisition.

Similarly, Krajcik et al. applied a quasi-experimental approach to measuring problem-solving abilities in science education [36]. Their research concluded that students taught through PBL exhibited enhanced PSS and retained science concepts more effectively than their peers in traditional learning settings. Moreover, Liao et al. analyzed PBL's role in cultivating critical thinking. Integrating pre- and post-assessment measures revealed notable advancements in students' critical thinking capabilities following PBL interventions [37]. Chen and Yang conducted a meta-analysis that synthesized data from multiple PBL studies, focusing on mathematics, a discipline often associated with student aversion [24]. They reported a consistent positive shift in students' attitudes toward mathematics when engaging in collaborative learning, confirming the affective benefits of this pedagogical approach. On the methodological front, the use of advanced statistical models has enriched PBL outcome analysis. For example, Tirado-Morueta et al. employed SEM to examine the complex relationships between PBL and SE [38]. Their study underscored the role of PBL in facilitating an active learning environment that fosters student motivation and engagement in mathematics.

These studies reflect the progressive trend toward validating PBL as an effective educational strategy. The current research, drawing on these methodological precedents and findings, aims to elucidate further the impact of PBL on enhancing SE in mathematics, thus contributing to the ongoing scholarly conversation.

## 3 Theoretical model

**Fig. 1:**
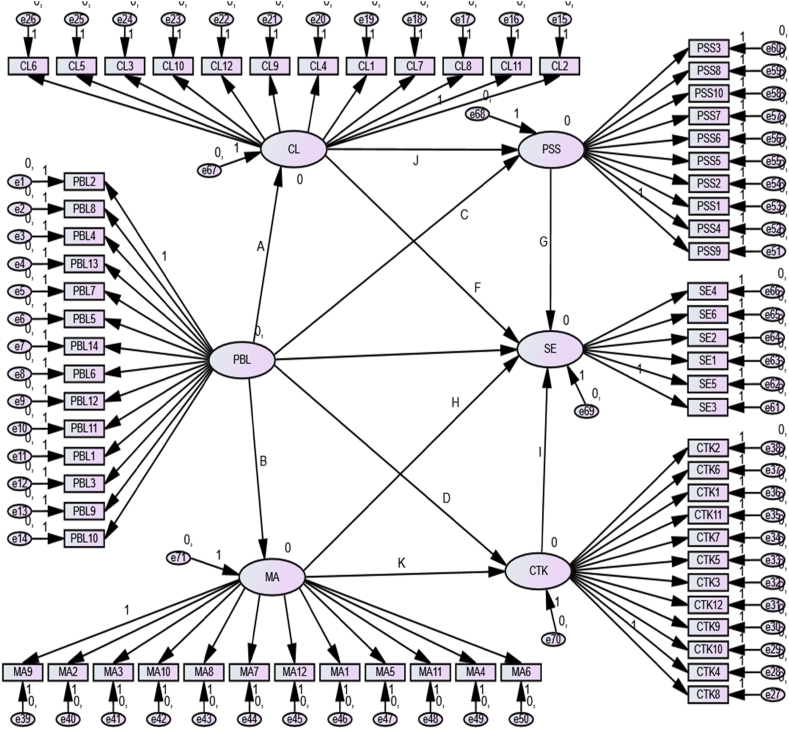
Research model and hypotheses.

The theoretical model of our study is built upon the foundational principles of PBL, which posits that students learn best when actively engaged in projects that require critical thinking, collaboration, and PSS essential for the 21st century. Each aspect of PBL, like MA, CL, CTK, and PSS, is hypothesized to be interrelated and to contribute to SE in math learning. This model is visualized in Fig. 1, where the arrows represent hypothesized relationships between the constructs. These relationships are informed by educational theories such as constructivism and situated learning, emphasizing the importance of context and practical application in learning. Each hypothesis reflects a theoretical linkage detailed in the subsequent sections, providing a rationale in the literature.

### 3.1 PBL and CL

Collaboration skills cannot be overstated because they are a treasure that can bring hands and minds together [39]. When people come together to work, they share ideas, help each other, and aim for a goal more significant than one person can reach alone. PBL, or PBL, is like a garden where teamwork skills grow [40]. This approach encourages students to collaborate and develop essential teamwork, communication, and mutual understanding skills for future endeavors in the broader world [41]. PBL encourages students to collaborate on authentic projects, mirroring the challenges they encounter in the real world outside of school [42]. This way of learning throws them into situations where they must talk things out, listen, and agree to get things done. It is like practicing for real life [43]. When students dive into PBL, they acquire more than just the lesson. They learn about each other—how to talk, listen, and put all their creative ideas together to make something great [37]. They learn that everyone has something to bring to the table, and by working together, they can solve even the trickiest problems [44].

However, PBL is not just about work. It is about building trust and making a space where everyone feels they belong and have a part to play [39]. When students work like this, they build skills that help them anywhere, such as being part of a team, being flexible, leading, and understanding different people [40]. The present study empirically proposes a hypothesis to examine the connection between PBL and CL, reflecting on these insights.

Hypothesis A PBL approach helps students to develop CL skills.

Hypothesis F CL helps students to engage in math learning.

### 3.2 PBL and MA

Math attitude (MA) is a multifaceted concept involving students' beliefs, emotions, and motivations toward mathematics, and it significantly influences their academic performance and engagement [45]. PBL transforms these attitudes by providing a platform where mathematical theories intersect with real-world problems, potentially nurturing a more positive outlook on mathematics [46]. As students engage with PBL, they are not only passive recipients of knowledge but also active participants in learning. This participatory approach brings mathematics to life, connecting abstract concepts to tangible experiences and applications. Such real-world integration naturally stirs students' curiosity and excitement, shifting their perception of math from a challenging subject to an accessible and vital discipline [47].

Moreover, PBL encourages students to own their learning journeys. As they navigate through project-based tasks with their peers, they cultivate collaborative problem-solving and critical thinking, a dynamic process that builds their confidence and reshapes their MA positively [48]. This enhanced MA fuels their CTK. When students view math enthusiastically and confidently, they are more likely to engage deeply with the content, apply higher-order thinking to solve problems, and effectively evaluate results [24]. The present study empirically proposes the following hypothesis to examine the connection between PBL and MA.

Hypothesis B PBL approach helps the students to develop a positive MA.

Hypothesis K A positive MA helps the students to develop CTK.

### 3.3 PBL and PSS

In the ever-evolving education landscape, addressing complex problems is paramount. Equipping our learners with robust PSS is non-negotiable as we steer our learners toward the future. PBL is a conduit for these skills, blending CTK with practical application to tackle real-world challenges [49]. This study aimed to illuminate the efficacy of PBL in nurturing PSS and fostering an environment where students can innovate and find creative solutions. PBL transcends conventional educational boundaries, ushering students into a realm where textbook theory meets tangible problem-solving practices [50]. As students immerse themselves in projects, they are called to dissect complex situations, consider multiple angles, and forge solutions grounded in critical thinking [42]. This educational model sparks intellectual curiosity and cultivates a collaborative mindset, as students must communicate their thoughts and unite with peers to pursue common goals [49]. Indeed, PBL is not a solitary endeavour but a collective endeavour in which collaborative learning intertwines with PSS. When students collaborate, they exchange strategies, challenge each other's thinking, and collectively navigate the problem-solving process. This interaction strengthens individuals’ collective problem-solving abilities, highlighting the symbiotic relationship between collaboration and problem-solving. The present study, building on these insights, empirically proposes a hypothesis to examine the connection between PBL and the development of PSS**.**

Hypothesis C PBL help the students to develop PSS.

Hypothesis J CL skills help the students to be involved in PSS.

### 3.4 PBL and CTK

CTK is the bedrock for nurturing lifelong learners and conscientious members of society [51]. PBL is a method and a transformative experience that instills these skills by challenging students with tasks that require deep analysis, varied perspectives, and well-reasoned conclusions [50]. Within PBL active learning environments, students transition from passive information absorption to active exploration and connection-making. They learn to navigate complex issues, engage in logical reasoning, and practice discernment, fostering an analytical and creative mindset [52]. PBL cultivates critical thinking and intertwines it with problem-solving, pushing students to question, evaluate, and devise solutions independently [53]. Critical thinking development through PBL has a ripple effect, reaching beyond individual tasks to enhance students' engagement in math learning. As they develop CTK, students are better equipped to engage with mathematical concepts; they can interrogate problems, understand mathematical arguments, and apply logic to various scenarios. This engagement is not a byproduct but a direct outcome of PBL's enriched critical thinking [54]. Our study proposes a hypothesis to examine the connection between PBL and CTK development empirically.

Hypothesis D PBL approach helps the students to develop CTK skills.

Hypothesis I CTK help the students to engage in math learning.

### 3.5 PBL and students’ engagement

The traditional education landscape often features a teacher-centric approach, with students serving as passive knowledge recipients [55]. The sociocultural perspective has challenged this model, underscoring the significance of active participation within learning communities, where cultural and historical practices enrich the learning experience [56]. Here, PBL has distinguished itself by actively valuing students' abilities to construct knowledge and meaning [57]. PBL is not just an instructional method but a catalyst for a paradigm shift from passive learning to active engagement. It aligns with the demands of the professional world, where collaborative endeavors are the norm [58]. PBL, recognized for its effectiveness, champions student-centered learning by encouraging learners to engage deeply with content that resonates with their interests and stimulates active participation [59]. In contrast to direct instruction, PBL provides a dynamic environment in which essential skills are developed, interactions are nurtured, and material mastery is facilitated [60,61].

The collaborative spirit intrinsic to PBL also enhances students' engagement in math learning. When students tackle real-world problems collaboratively, their attitude toward math can transform from apprehension to appreciation. This engagement acts as a bridge, connecting individual skills development to a collective learning experience. This study proposes a hypothesis to empirically examine the connection between PBL and students' engagement in collaborative learning [85].

Hypothesis E PBL approach helps the students to engage in Math learning.

Hypothesis H A positive MA helps the students to engage in math learning.

Hypothesis G PSS helps the students to engage in math learning.

This study's dependent variable was "SE in math learning," while the independent variables were PBL: CL, MA, PSS, and CTK. The hypotheses outlined in the study aim to establish significant relationships between the independent variables (PBL, CL, MA, PSS, and CTK) and the dependent variable (SE in math learning). These hypotheses provide a framework for investigating the relationships between the independent and dependent variables. This contributes to a deeper understanding of the impact of PBL on SE in math learning.

## 4 Methodology

Many educational institutions, including Islamabad public schools, have embraced PBL. This study seeks to empirically investigate the effectiveness of PBL in engaging students in math learning, aiming to develop a model for assessing its impact. The research focuses on four key factors: CL, CTK, PSS, and MA, which collectively contribute to SE in math learning. High school students were given questionnaires to measure these factors. The questionnaire consisted of two main sections: the first section assessed the independent factors of PBL (CL, PSS, CTK, and MA), while the second section measured the dependent factor (SE). A purposive sampling technique was employed to select a sample of 600 students from ten schools in Islamabad where PBL had been implemented in mathematics teaching. This method was selected because it allowed us to deliberately choose schools that were actively utilizing PBL, ensuring that the participants had direct exposure to the pedagogical approach being studied. The use of purposive sampling was essential to the study's objectives, as the focus was on evaluating the impact of PBL on developing 21st-century skills and SE. Random sampling would not have guaranteed that all participants were from schools implementing PBL, which was crucial for collecting relevant and valid data for this investigation.

The questionnaire used in this study was validated for both face and content validity by two experts in the field. A 5-point Likert-type scale (ranging from "strongly disagree" to "strongly agree") was employed to assess students' perceptions of 21st-century skills (CL, PSS and CTK) and their engagement in mathematics learning.

CFA was conducted using SEM-AMOS to ensure the measurement model's reliability and validity. Several types of validity, such as convergence and discriminant validity, were measured following the guidelines from Hair et al. (Please see Table 3).

Following Shiau et al.'s methodology, the analysis was conducted in two phases: the first phase assessed the measurement model's structure, convergence, and discriminant validity, while the second phase examined the structural model [84].

**Fig. 2:**
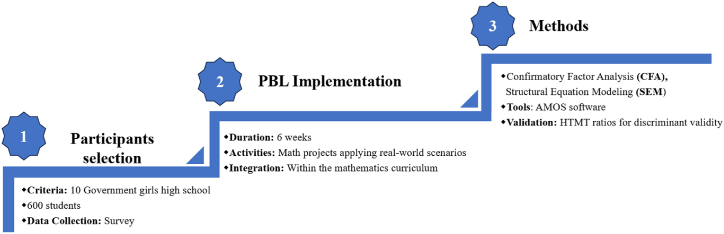
Methodology overview for PBL study.

By utilizing SEM, this study explores the interplay between the dimensions of PBL (CL, CTK, PSS) and MA, and their collective influence on SE, providing a comprehensive understanding of these dynamics (see Fig. 2). The complexity of these relationships necessitated the use of SEM, which allows for the simultaneous analysis of both direct and indirect effects between multiple latent variables, such as CL, PSS, CTK, and SE. CFA a component of SEM, was employed to validate the measurement model, ensuring that the observed data accurately represented the latent constructs. Traditional methods like regression or ANOVA would not allow for such comprehensive modeling of these complex relationships.

### 4.1 Measurement instruments

The present study implemented a collaboration scale derived from TIBI's (2015) work to appraise students' collaborative abilities. It included a questionnaire comprising 12 statements, each following a five-point Likert-style scale. A questionnaire of ten statements was also used to examine students' problem-solving capabilities. Moreover, to assess the students' creative and critical skills, a questionnaire was adopted from Gelerstein, Yoon, Sumarni, and Kadarwati [[62], [63], [64]]. Ventista also employed the same questionnaire in his study [65]. The scale's reliability was assessed using Cronbach's alpha test, which returned a reliability score of 0.76. Furthermore, the scale developed by Aladağ and later updated by Eskici et al. was employed to gauge the student's attitude toward mathematics [66,67]. The scale was also implemented in a research study by Mazana et al. [68]. Finally, we adopted a six-item questionnaire from Almulla's study to measure SE in learning processes [58].

### 4.2 Sample characteristics

The purposive sampling technique was used to collect data from ten public schools in Islamabad that used PBL as an instructional tool. The schools are located in the G-10 sector, G-9 sector, G-11 sector, F-7 sector, and G10-4 sector of Islamabad. Six hundred students were selected from these ten schools for data collection. This study's participants were all female high school students because it was conducted in only girls' schools. Informed consent was obtained from all participants before data collection. Participants provided written consent, and the study was approved by the Research Ethics Committee of Zhejiang Normal University. Ethics approval was granted under reference number ZSRT2024191.

## 5 Results

It is crucial to assess the dimensions of the constructs before using SEM, primarily to ensure that the scale shows one-dimensional results when items are evaluated independently. The research utilized exploratory factor analysis (with varimax rotation) on 66 measured items. The analysis was considered successful and statistically significant, with all the items showing factor loadings greater than 0.5. Babin and Anderson suggested that loadings of 0.30 are minimally acceptable, 0.40 are more substantial, and 0.50 or higher are considerably significant. Additionally, the dataset's validity and the sample size's sufficiency were verified using the Kaiser-Meyer-Olkin (KMO) and Bartlett's tests. The KMO value was recorded as 0.808, surpassing the acceptable threshold of 0.5, and Bartlett's test showed significant results, supporting the comprehensiveness of the variable analysis.

SEM was selected for this investigation to evaluate the effect of the independent variables on the study's dependent variable. The selection was based on the strength of SEM, which includes a confirmatory approach to data analysis, including latent and observed variables and the ability to model complex variable relationships while estimating direct and indirect effects within the research.

### 5.1 Confirmatory factor analysis

CFA is an essential step in the SEM process [69]. It was implemented before constructing the structural model to confirm that the constructs aligned appropriately with the data. The purpose of CFA is to assess the compatibility of the data with a preconceived measurement model [70]. This study conducted both initial and modified CFAs for the test constructs. Modifications to the CFA models were necessary because the original CFAs did not meet the predefined standards. The results of the initial CFA fitness indices for all the constructs are displayed in Table 1.

**Table 1:** Goodness of model fit and reliability.

Measure	Estimate	Threshold	Interpretation
CMIN	2467.463	–	–
DF	2064	–	–
CMIN/DF	1.195	Between 1 and 3	Excellent
CFI	0.984	>0.95	Excellent
SRMR	0.028	<0.08	Excellent
RMSEA	0.018	<0.06	Excellent
IFI	0.985	>0.95	Excellent
NFI	0.912	>0.90	Excellent
RFI	0.909	>0.90	Excellent
TLI	0.984	>0.95	Excellent

Predictor	CR	AVE	MSV	MaxR(H)
PBL	0.951	0.583	0.406	0.951
CL	0.942	0.574	0.304	0.942
MA	0.937	0.553	0.301	0.937
CTK	0.944	0.586	0.401	0.944
PSS	0.938	0.601	0.372	0.938
SE	0.888	0.569	0.406	0.889

Note: CR = Composite Reliability; AVE = Average Variance Extracted; MSV = Mean Shared Values; PBL = Project-Based Learning; CL = Collaborative Learning; MA = Math Attitude; CTK = Critical Thinking Skills; PSS = Problem-Solving Skills; SE = Student Engagement.

Model fit and reliability were assessed based on the SEM-AMOS model results. The model fit indices indicate a good fit of the data to the hypothesized model. The chi-square test statistic (CMIN) value was 2467.463, with 2064 degrees of freedom (DF), resulting in a CMIN/DF ratio of 1.195, which falls within the acceptable range of 1–3. This finding suggested that the model provided an excellent fit for the data. Several other fit indices were also evaluated. The comparative fit index (CFI) was 0.984, exceeding the recommended threshold of 0.95, indicating an excellent fit. The standardized root mean square residual (SRMR) was calculated to be 0.028, below the threshold of 0.08, further supporting an excellent model fit. Additionally, the root mean square error of approximation (RMSEA) yielded a value of 0.018, below the recommended cutoff of 0.06, indicating an adequate fit of the model to the data.

Furthermore, other fit indices, including the incremental fit index (IFI), normed fit index (NFI), relative fit index (RFI), and Tucker-Lewis index (TLI), were also assessed. The values obtained for these indices (IFI = 0.985, NFI = 0.912, RFI = 0.909, TLI = 0.984) exceeded the respective thresholds of 0.95 and 0.90, indicating excellent fit.

The first predictor, PBL, demonstrated a high composite reliability (CR) of 0.951, indicating solid internal consistency. Its average variance extracted (AVE) is 0.583, which explains approximately 58.3 % of the variance in its corresponding construct. The maximum shared variance (MSV) is 0.406, suggesting significant overlap with the other predictors, while the maximum redundancy (MaxR) is 0.951, suggesting that PBL strongly influences its respective construct. Similarly, CL exhibited a high CR value of 0.942, indicating that it was a reliable measure. Its AVE is 0.574, which accounts for approximately 57.4 % of its variance. The MSV is 0.304, suggesting moderate overlap with other predictors, and the MaxR is 0.942, indicating a strong influence on its construct. Moving on to MA, the CR value was 0.937, indicating good internal consistency. Its AVE is 0.553, indicating that it explains approximately 55.3 % of the variance in its construct. The MSV was 0.301, suggesting moderate overlap with the other predictors. The MaxR is 0.937, signifying a substantial influence on its construct; another predictor demonstrates a CR value of 0.944, reflecting high internal consistency. Its AVE is 0.586, implying that it explains approximately 58.6 % of the variance in its construct. The MSV was 0.401, suggesting moderate overlap with the other predictors. The MaxR is 0.944, indicating that it strongly influences the construct. The PSS exhibited a CR value of 0.938, indicating good internal consistency. Its AVE is 0.601, indicating that it explains approximately 60.1 % of the variance in its construct. The MSV was 0.372, suggesting a moderate overlap with the other predictors. As a result, MaxR was 0.938, which indicates that its construct is strongly influenced. The last predictor, SE, has a CR value of 0.888, indicating good internal consistency—an AVE of 0.569 accounts for nearly 56.9 % of the construct's variance. The MSV is 0.406, which shows a reasonable correlation with the other predictors—the MaxR of 0.889 significantly impacts its construct.

The results indicate that the model is in good harmony with the collected data. This finding confirms the credibility and validity of factors linked to cooperation and communication in learning. An impressively high reliability coefficient (Cronbach's alpha) of .947 boosts the model's competence, which signifies solid internal consistency of the analyzed factors and items. Furthermore, factor loadings of 0.7 or above indicate a commendable reliability level, and a composite confidence value exceeding 0.70 signals a robust model. These observations underscore that the SEM-AMOS model applied in this study effectively encapsulates the relationships among the variables of concern, thus offering a trustworthy and valid framework for grasping the factors that influence cooperation and communication in learning (see Table 1).

### 5.2 Analysis of the measurement model

**Fig. 3:**
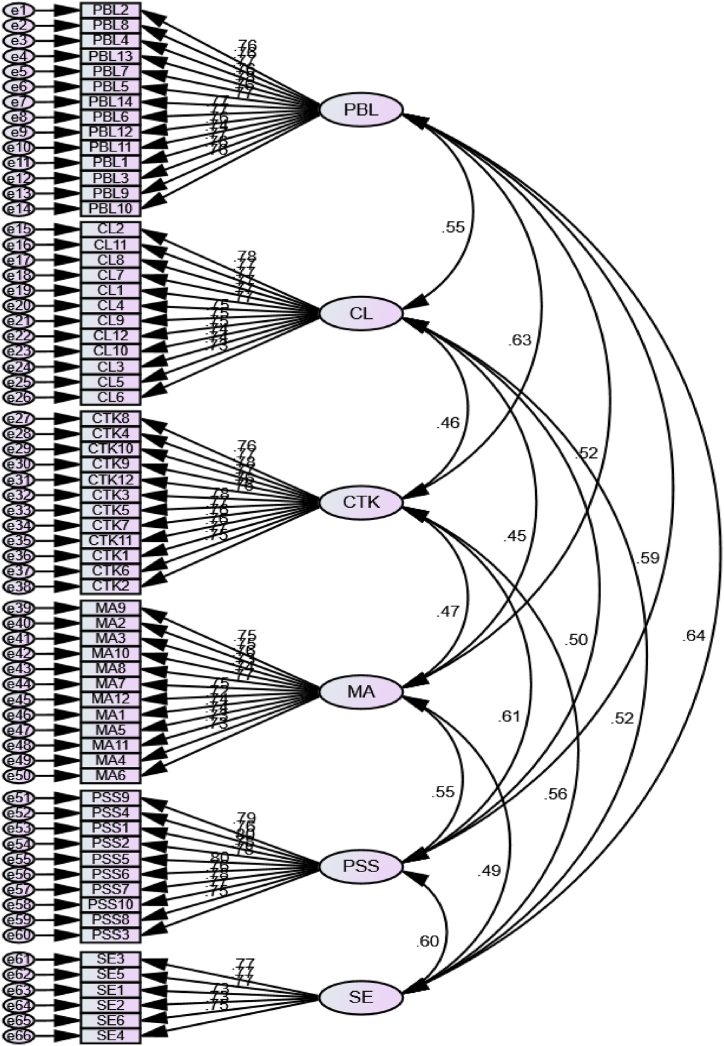
Hypothesized initial measurement model (pooled CFA) of the associations between the constructs of the study.

In this research, the researcher primarily relied on the SEM-AMOS approach to analyze the gathered data statistically. With AMOS 23, the researcher performed a (CFA) to scrutinize the model's validity. It included a detailed assessment of several parameters, such as convergent validity, one-dimensionality, consistency, and discriminant validity. Adhering to the guidelines proposed by Ong and Puteh, we resorted to the highest probability estimation method for evaluating our model [71]. We incorporated a range of fit indices into our evaluation process to ensure a comprehensive assessment. These included the chi-square test, normed chi-square test, normed fit index (NFI), relative fit index (RFI), Tucker–Lewis index (TLI), comparative fit index (CFI), incremental fit index (IFI), parsimonious goodness-of-fit index (PGFI), root mean square residual (RMR), and root mean square error of approximation (RMSEA). The evaluation of our model led to specific results, which are systematically documented in Table 1 and further visually represented in Fig. 3 for a better understanding of the measurement model.

### 5.3 Validity and reliability of the measurement model

Discriminant validity involves carefully examining the degree of distinction, including various indicators related to different constructs. A close examination of the AVE values showed that all values surpassed the 0.50 cutoff, with a p-value of .001, indicating a favorable accord of discriminant validity across all frameworks analyzed [72]. Furthermore, Rönkkö & Cho have clarified that the correlation among elements within a construct should not exceed the square root of the shared variance average for any of the other constructs [73]. Composite reliability values are displayed, falling convincingly within the accepted range of 0.70 or more. In parallel, the values for Cronbach's alpha fall within the recommended range of 0.70 or higher, and the AVE values meet or exceed the advised threshold of 0.50. It confirms that the overall factor loading is above the significant benchmark of 0.50. (See Fig. 3 and Table 3 for more details). The results presented in Table 2 suggest that the constructs measured are statistically significant at a p-value of less than 0.05, as indicated by their parameter estimates.

**Table 2:** Factor loadings, composite reliability, and AVE.

Construct	Items	Factor Loadings
PBL Alpha = 0.951 AVE = 0.583 CR = 0.951 MSV = 0.406 MaxR(H) = 0.951	Solving problems with friends made the problem-solving process easier to manage.	0.758
	I gained more confidence in solving mathematics problems through a PBL approach because of help from friends and teachers.	.764
	The challenge of solving the problem task kept me going and thinking.	.765
	I enjoyed working on math projects with my friends	.763
	PBL helped me figure out mathematical problems that do not appeal to me	.781
	PBL changed my perception of math learning	.761
	PBL enhanced the skills required for solving mathematical problems	.767
	PBL helped me to be more in the learning process	.770
	PBL helped me to develop analytical and PSS in mathematics	.768
	PBL changed my approach to mathematics	.756
	I prefer this approach (PBL) to solve mathematics problems rather than textbooks.	.745
	This PBL approach makes mathematics more interesting and challenging.	.768
	PBL significantly enhanced my understanding of a mathematical concept	.760
	PBL helped me to adapt to challenges or obstacles in understanding mathematical concepts	.763
CL Alpha = 0.942 AVE = 0.574 CR = 0.942 MSV = 0.304 MaxR(H) = 0.942	The discussion forum participants provided helpful feedback to each other regarding the course project.	0.776
	I kept the necessary materials and information for the project for myself	.770
	I made a unique contribution to the joint effort.	.773
	I tried to discuss concepts being learned with others.	.767
	I felt responsible for observing other participants' work.	.773
	Sometimes, I tried to give direction to the group's work.	.769
	I tried to compliment people when I liked something they had done.	.752
	We monitored each other's work to ensure the high quality of the course project.	.751
	I felt we depended on each other while working on the course project.	.750
	From time to time, I checked for other participants' understanding of the learned Materials	.740
	The discussion forum participants tried to find ways to solve group problems.	.739
	The discussion forum participants provided helpful feedback to each other regarding the course project.	.734
MA Alpha = 0.937 AVE = 0.553 CR = 0.937 MSV = 0.301 MaxR(H) = 0.937	I have usually enjoyed studying mathematics in school	0.752
	I like to solve new problems in mathematics	.754
	I would prefer to do an assignment in mathematics than to write an essay	.764
	I like mathematics	.732
	I am happier in a mathematics class than in any other class	.738
	Mathematics is a fascinating subject	.773
	Winning a prize in mathematics would make me feel unpleasantly conspicuous	.745
	I am comfortable expressing my ideas on finding solutions to a complex problem in mathematics.	.720
	I am comfortable answering questions in mathematics class.	.738
	Mathematics is dull	.744
	When a math problem arises that I can’t immediately solve, I stick with it until I have a solution	.731
	Math problems challenge me I can’t understand immediately	.728
CTK Alpha = 0.944 AVE = 0.586 CR = 0.944 MSV = 0.401 MaxR(H) = 0.944	PBL helped me to connect mathematical concepts to real-world situations, enhancing my critical thinking.	0.757
	I noticed increased curiosity and interest in understanding the 'why' behind mathematical rules and formulas.	.768
	Through PBL, I developed a systematic approach to solving problems and making decisions based on logical reasoning.	.781
	This approach has taught me the importance of viewing mathematical problems from multiple angles before deciding on a solution.	.769
	The iterative nature of project work has improved my patience and thoroughness in addressing complex mathematical tasks.	.758
	The hands-on nature of PBL made me more persistent in solving mathematical challenges.	.761
	I feel more confident in my ability to analyze and break down mathematical problems because of my PBL experiences.	.777
	Collaborating with peers on projects led me to appreciate different perspectives and strategies for tackling mathematical questions.	.767
	This approach will help me tackle unfamiliar and challenging problems in the future.	.763
	PBL encouraged me to question the standard methods and explore alternative solutions in mathematics.	.762
	This approach helps me to think of multiple solutions	.767
	I found myself more actively engaged in solving complex problems during PBL activities.	.753
PSS Alpha = 0.938 AVE = 0.601 CR = 0.938 MSV = 0.372 MaxR(H) = 0.938	PBL equipped me with practical PSS applicable beyond the classroom	0.785
	hands-on learning in math projects made problem-solving more engaging and effective	.758
	PBL allowed me to grow as a problem-solver in mathematics	.795
	PBL showed me the real-life applications of math problem-solving	.782
	PBL enhanced my confidence in tackling complex math problems	.779
	Working on math projects with peers helped me improve my teamwork and collaboration skills	.797
	PBL sparked my interest in mathematics problem-solving	.757
	Through PBL, I learned to reflect on my problem-solving strategies and improve them	.779
	I discovered my creative side when solving math problems through PBL	.767
	I can relate PBL to my ability to solve real-world math problems	.750
SE Alpha = 0.887 AVE = 0.569 CR = 0.888 MSV = 0.406 MaxR(H) = 0.889	PBL makes me eager to explore and apply math in different contexts	0.771
	PBL helps me see the practical relevance of math in my daily life.	.767
	PBL in math encourages me to participate in class actively.	.772
	Through PBL, I feel more connected to the mathematical concepts we are studying.	.733
	PBL in math class sparks my curiosity and interest in the subject.	.731
	PBL has increased my engagement with mathematics, making it a more enjoyable experience	.752

a = CR = Composite Reliability; AVE = Average variance extracted; MSV = Mean Shared Values.

Note: CR = Composite Reliability; AVE = Average Variance Extracted; MSV = Mean Shared Values; PBL = Project-Based Learning; CL = Collaborative Learning; MA = Math Attitude; CTK = Critical Thinking Skills; PSS = Problem-Solving Skills; SE = Student Engagement.

**Table 3:** Discriminant validity (HTMT ratios).

	PBL	CL	MA	CTK	PSS	SE
PBL						
CL	0.552					
MA	0.518	0.449				
CTK	0.633	0.458	0.47			
PSS	0.593	0.502	0.548	0.61		
SE	0.638	0.521	0.495	0.564	0.6	

Correlation (fornell larcker criteria)						
	PBL	CL	MA	CTK	PSS	SE
PBL	**0.764**					
CL	0.551∗∗∗	**0.758**				
MA	0.518∗∗∗	0.449∗∗∗	**0.743**			
CTK	0.633∗∗∗	0.456∗∗∗	0.471∗∗∗	**0.765**		
PSS	0.592∗∗∗	0.500∗∗∗	0.549∗∗∗	0.610∗∗∗	**0.775**	
SE	0.637∗∗∗	0.520∗∗∗	0.494∗∗∗	0.564∗∗∗	0.601∗∗∗	**0.754**

**Note:** HTMT = Heterotrait-Monotrait ratio of correlations; PBL = Project-Based Learning; CL = Collaborative Learning; MA = Math Attitude; CTK = Critical Thinking Skills; PSS = Problem-Solving Skills; SE = Student Engagement.

To determine whether the predictors are more strongly related to their corresponding constructs than other constructs, the HTMT ratios need to be less than one. This finding indicates that predictors are more strongly associated with their corresponding constructs than others. Table 3 shows that all the HTMT ratios fall below the threshold of 1, suggesting acceptable discriminant validity. The HTMT ratio between PBL and CL is 0.552, indicating that the correlation between PBL and CL is lower than between PBL and its construct. Similarly, the HTMT ratio between MA and CTK was 0.47, indicating that the correlation between MA and CTK was lower than between MA and its construct. The HTMT ratios in Table 3 provide evidence of discriminant validity. It suggests that the predictors in the study are more strongly related to their respective constructs than to other constructs, supporting the distinctiveness of the measured variables.

Additionally, we employed the Fornell-Larcker criterion to further assess discriminant validity. Specifically, we ensured that the square root of the AVE for each construct (see Table 3, fornell larcker criteria) was greater than the correlations between that construct and all others. This comparison confirms that the constructs are distinct from one another. Furthermore, the squared correlations between each pair of constructs were lower than the corresponding AVE values, supporting the discriminant validity of the model.

The correlation coefficients indicate the strength and direction of the relationships between the variables. The correlation coefficient between PBL and CL is 0.551, which is statistically significant (∗∗∗), indicating a moderately strong positive relationship between these two predictors. Similarly, the correlation coefficient between PBL and MA is 0.518, which is also significant, suggesting a moderate positive relationship between them. Additionally, the correlation coefficient between PBL and CTK is 0.633, indicating a relatively strong positive relationship, while the correlation coefficient between PBL and PSS is 0.592, indicating a moderately strong positive relationship. The correlation coefficient between PBL and SE is 0.637, suggesting a relatively strong positive relationship. The other correlation coefficients in the table follow a similar pattern, indicating significant positive relationships between the corresponding predictors. These patterns of association underscore the interconnectedness and relevance of PBL to collaborative learning, critical thinking, PSS, and SE, highlighting its importance and impact in educational settings.

### 5.4 Structural model analysis

**Fig. 4:**
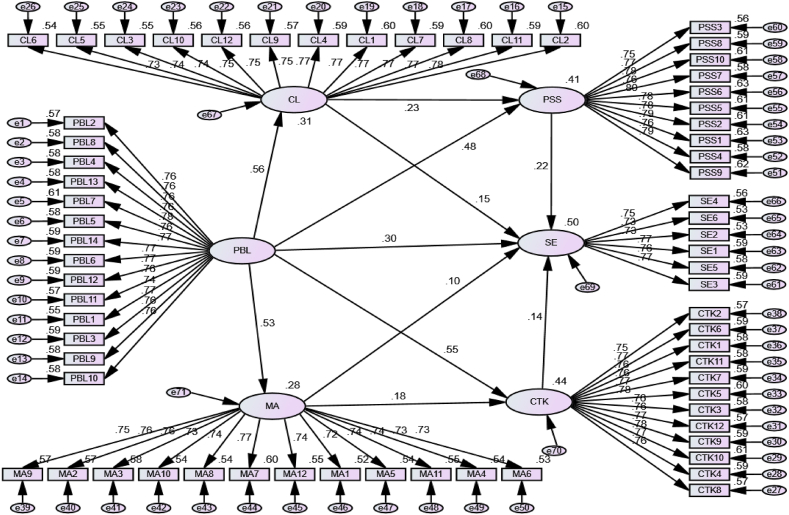
Structural model and hypothesis testing.

Structural model analysis examines SE through the influences of PBL, CL, PSS, and CTK. Fig. 4 and Table 4 compare these findings in the hypothesis examination discourse. Table 4 shows the results derived from hypothesis testing. The table shows the predictor variables, the relationships scrutinized, the outcome metrics, and the corresponding approximations, standard deviations (SDs), critical ratios (CRs), probability values (P), and R-squared values. The hypotheses have been tagged A through K.

**Table 4:** Hypothesis testing.

Hypothesis	Predictor	Relationship	Outcome	Estimate^a^	SE.	CR.	P	R-Square
H-A	PBL	--->	CL	0.560	0.050	12.340	∗∗∗	0.31
H-B	PBL	--->	MA	0.531	0.043	11.591	∗∗∗	0.28
H-D	PBL	--->	CTK	0.550	0.053	11.294	∗∗∗	0.44
H-K	MA	--->	CTK	0.178	0.048	4.289	∗∗∗	
H-C	PBL	--->	PSS	0.480	0.053	9.967	∗∗∗	0.41
H-J	CL	--->	PSS	0.231	0.044	5.254	∗∗∗	
H-E	PBL	--->	SE	0.295	0.054	4.835	∗∗∗	0.5
H-I	CTK	--->	SE	0.139	0.038	2.967	0.003	
H-G	PSS	--->	SE	0.222	0.037	4.796	∗∗∗	
H-F	CL	--->	SE	0.145	0.035	3.347	∗∗∗	
H-H	MA	--->	SE	0.097	0.039	2.330	0.02	

Note: CR = Critical Ratio; CL = Collaborative Learning; MA = Math Attitude; PSS = Problem-Solving Skills; CTK = Critical Thinking Skills; SE = Student Engagement in Math Learning.

a Standardized Regression Weights.

Based on hypothesis A, the predictor variable PBL was significantly correlated with CL (β = 0.560, t = 12.340, p < 0.001). This points toward a significant effect of PBL on collaborative learning. Turning to hypothesis B, a significant positive correlation was noted between PBL and MA (β = 0.531, t = 11.591, p < 0.001). This finding suggested that PBL significantly affects MA. Hypothesis C examines the relationship between PBL and PSS (β = 0.480, t = 9.967, p < 0.001). The outcomes exhibit a strong positive link, indicating that the PBL method has a meaningful effect on PSS.

Hypothesis D revealed a significant positive correlation between PBL and CTK (β = 0.550, t = 11.294, p < 0.001), implying that the PBL method significantly impacts CTK. Hypothesis E investigates the relationship between PBL and SE (β = 0.295, t = 4.835, p < 0.001). The outcomes present a significant positive correlation, suggesting that the PBL method is crucial for enhancing SE in learning. Switching to hypothesis F, a significant positive correlation was found between CL and SE (β = 0.145, t = 3.347, p < 0.001), indicating that collaborative learning profoundly impacts SE. Hypothesis G studies the link between PSS and SE, unveiling a significant positive correlation (β = 0.222, t = 4.796, p < 0.001). These findings show that PSS significantly affects SE. Hypothesis H establishes a significant positive relationship between MA and SE (β = 0.097, t = 2.330, p = 0.020), suggesting that a positive attitude toward Math influences students' engagement in learning activities.

For hypothesis I, a significant positive correlation was detected between CTK and SE (β = 0.139, t = 2.967, p = 0.003), suggesting that CTK plays a pivotal role in influencing SE. Turning to hypothesis J, a significant positive correlation between CL and PSS is observed (β = 0.231, t = 5.254, p < 0.001), implying that collaborative learning substantially impacts PSS. For hypothesis K, a noticeable positive relationship between MA and SE (β = 0.097, t = 2.330, p = 0.020) emerges, indicating that MA significantly impacts SE.

These findings support hypotheses and unravel the significant links between the PBL approach and the other variables encompassing collaborative learning, disciplinary subject learning, and authentic learning.

Additionally, to address the role of mediators, the results show partial mediation in several paths. For instance, CL, PSS, MA, and CTK mediate between PBL and SE, as demonstrated by the indirect effects in Table 5. However, these mediators do not fully explain the relationship between the variables, as the direct effects of PBL on SE remain significant in most cases, suggesting partial mediation rather than full mediation [86].

**Table 5:** Conditional specific indirect effects for mediation.

Parameter	Estimate	Lower	Upper	P	Results
PBL →CL→SE	0.071	0.029	0.116	0.002	Accepted
PBL →PSS→SE	0.094	0.051	0.142	0.001	Accepted
PBL →MA→SE	0.045	0.002	0.087	0.036	Accepted
PBL →CTK→SE	0.067	0.022	0.114	0.004	Accepted
PBL →CL→PSS→SE	0.025	0.012	0.043	0.001	Accepted
PBL →MA→CTK→SE	0.012	0.003	0.023	0.004	Accepted

Note: PBL = Project-Based Learning; CL = Collaborative Learning; MA = Math Attitude; CTK = Critical Thinking Skills; PSS = Problem-Solving Skills; SE = Student Engagement.

Table 5 displays the results of the conditional specific indirect effects for mediation. The table includes the parameters tested, along with their estimates, lower and upper bounds, p-values (P), and the corresponding results. Starting with the first parameter, PBL→ CL → SE, the estimate is 0.071, with lower and upper bounds of 0.029 and 0.116, respectively. The p-value is 0.002, indicating that this indirect effect is statistically significant and acceptable. For the following parameter, PBL → PSS → SE, the estimate is 0.094, with lower and upper bounds of 0.051 and 0.142, respectively. The p-value is 0.001, suggesting that a significant indirect effect is accepted. For the parameter PBL → MA → SE, the estimate is 0.045, with lower and upper bounds of 0.002 and 0.087, respectively. The p-value is 0.036, indicating that a significant indirect effect is accepted. Next, the parameter PBL → CTK → SE has an estimate of 0.067, with lower and upper bounds of 0.022 and 0.114, respectively. The p-value is 0.004, indicating that a significant indirect effect is accepted. For PBL → CL → PSS → SE, the estimate is 0.025, with lower and upper bounds of 0.012 and 0.043, respectively. The p-value is 0.001, suggesting that a significant indirect effect is accepted. Finally, for the parameter PBL → MA → CTK → SE, the estimate is 0.012, with lower and upper bounds of 0.003 and 0.023, respectively. The p-value is 0.004, indicating that a significant indirect effect is accepted.

In summary, Table 4 provides the conditional indirect effects of PBL on SE through CL, PSS, MA, and CTK. The significant indirect effects demonstrate that PBL indirectly impacts SE through these variables, reinforcing their role as partial mediators in the model. The results suggest that while the mediators play a significant role, the direct effects of PBL on SE persist, confirming partial mediation rather than full mediation.

## 6 Discussion and implications

The main objective of this study was to introduce a novel approach utilizing PBL (PBL) to enhance CL, PSS, CTK, and MA among students in high secondary schools. This study represents a pioneering effort in implementing a PBL approach for high school students. The proposed model investigates the relationships between the PBL approach and the abovementioned factors (CL, PSS, CTK, MA, and SE), as illustrated in Fig. 2. By applying the PBL approach in high school settings, this research emphasizes the importance of CL, PSS, CTK, and MA in engaging students in mathematics education. Ultimately, a PBL strategy prepares learners with the essential tools to navigate real-world problems and meet intended learning objectives, supported by previous research [6,24,74].

The findings of this research provide us with a helpful perspective on the interplay between various elements influencing student participation in learning. The process of hypothesis testing revealed multiple meaningful relationships, furthering our knowledge of SE determinants. Additionally, our analysis revealed partial mediation in several paths. Specifically, CL, PSS, MA, and CTK act as partial mediators between PBL and SE, as demonstrated by the indirect effects in Table 5. While these mediators significantly contribute to explaining the relationship, the direct effects of PBL on SE remain significant, indicating partial mediation rather than full mediation. This highlights that although the mediating variables play an important role, PBL continues to have a direct influence on SE.

The findings initially supported hypothesis A, suggesting a substantial positive correlation between PBL and CL. This conclusion aligns with previous studies [75], emphasizing the effectiveness of PBL in fostering student interaction and encouraging active participation in the learning process.

Moreover, hypothesis B exhibited a considerable positive correlation between PBL and MA, a result that aligns with the findings of Demir and Önal highlighting the significant role of PBL in nurturing a positive mathematical disposition, consequently inspiring a more significant engagement in mathematics education [76]. The outcome of hypothesis D indicated a substantial positive correlation between PBL and CTK. This observation concludes that PBL strategies can expedite profound learning in discipline-specific subjects [60]. Involving students in genuine, real-world problem-solving activities using PBL can amplify their comprehension and practical application of subject-specific knowledge and skills. The outcome of hypothesis C pointed to a considerable positive correlation between PBL and student PSS. This conclusion resonates with recent research emphasizing student satisfaction's tight correlation with PBL-driven learning experiences [77]. The practical, student-centered nature of PBL develops a sense of accomplishment and ownership, increasing satisfaction levels.

Additionally, hypothesis H's outcome revealed a significant positive correlation between MA and SE. This result aligns with that of Gjicali and Lipnevich's research, indicating the substantial influence of a positive MA on SE in mathematics education [78]. A positive disposition toward math is critical in fostering active participation and internal motivation among students [79].

The findings of this study offer a novel contribution to the literature on PBL by providing empirical evidence of its specific impact on the development of 21st-century skills and SE in mathematics learning. Unlike previous studies, which have largely focused on general educational outcomes, this study uniquely investigates these relationships within the context of public schools in Pakistan. By focusing on female students in government-run girls' schools, this research addresses a critical gap in gender-specific educational interventions, offering new perspectives on the effectiveness of PBL in such environments. Additionally, the study’s use of CFA and SEM provides a methodological advancement by offering a validated model that can be replicated or extended in future research.

While this study offers valuable insights, several limitations should be acknowledged to provide a more comprehensive understanding of the findings. The data in this study are based on student self-reports, which may be subject to biases or inaccuracies due to subjective perceptions. Future studies could incorporate objective measures, such as observational data, teacher assessments, or performance metrics, to validate and complement self-reported findings. The six-week duration of the PBL intervention may not be sufficient to capture the long-term effects of PBL on SE and learning outcomes. To address this, future research should adopt longitudinal designs that examine the sustained impact of PBL over longer periods. This study exclusively focused on girls' schools, which limits the generalizability of the findings to other school settings, such as coeducational or boys' schools. While this study focuses on the direct relationships between PBL and key constructs such as CL, PSS, CTK, and SE, future research could explore mediated or moderated pathways. For instance, researchers could investigate whether certain factors, such as motivation, teacher support, or classroom environment, act as mediators or moderators that influence the strength of the relationship between PBL and student outcomes. Such analyses could provide a deeper understanding of the mechanisms through which PBL affects learning and engagement.

By addressing these limitations, future studies can provide a more nuanced understanding of the impact of PBL on SE and learning, enhancing the robustness and generalizability of the findings.

The findings of this study underscore the significance of PBL, MA, and student satisfaction in driving SE in learning. Educators should aim to create learning environments that stimulate collaboration, positive attitudes, and satisfaction to cultivate active SE and optimize learning outcomes. Several authorities in the field have thoroughly documented this conclusion, with Tsybulsky and Muchnik-Rozanov characterizing the PBL approach as "constructivist." For example, Craig et al. stressed it as one of the finest exemplars of a constructivist learning environment [80,81,83].

Furthermore, the design of higher education courses, including online learning and massive open online courses, often focuses on constructivism-centered practices and tasks that foster collaboration and SE in learning [82]. The present research model on the impact of the PBL approach reveals that it improves CL, PSS, CTK, and MA, enhancing SE in math learning. Consequently, schools should actively encourage teachers to adopt the PBL approach and make students aware of the significant educational benefits they can achieve by utilizing this approach. The study provides empirical evidence of the effectiveness of the PBL approach, specifically in promoting CL, PSS, CTK, MA, and SE, and of its positive impact on student learning (refer to Table 3 for the hypotheses). Based on these results, two critical implications can be drawn.

• Schools should invest in professional development to equip teachers with the necessary skills to implement PBL effectively. Structured PBL training can help teachers foster critical thinking and collaboration among students.
• Educational policymakers should integrate PBL into curricula, particularly in math education, to promote 21st-century skills. Schools should encourage the use of real-world projects to enhance student engagement and learning outcomes.

## 7 Conclusion

In conclusion, the findings from the SEM-AMOS analysis provide valuable insights into the relationships between the predictors and their respective constructs. The results indicate that all the predictors (PBL, CL, MA, CTK, PSS, and SE) demonstrate solid internal consistency and explain significant variance in their corresponding constructs. However, there is a moderate overlap among the predictors, suggesting potential interrelationships. These findings contribute to understanding how the PBL approach can enhance CL, MA, CTK, and PSS among students in high secondary schools. Previous studies, such as those by Rehman et al. and Darmuki et al., have similarly highlighted the effectiveness of PBL in fostering 21st-century skills [3,6,83]. The significant correlations observed in this study align with theories proposed by Chistyakov et al. and Almazroui, underscoring the transformative potential of PBL in educational settings [4,9].

This research supports the importance of implementing the PBL approach in high school settings to engage students in mathematics learning and prepare them for real-life challenges. This study represents a significant contribution to the field, paving the way for future research into the effectiveness of the PBL approach in various educational contexts. Future studies should incorporate teachers' and principals' viewpoints on applying PBL in educational settings.

Additionally, educators should consider incorporating novel teaching methodologies specific to mathematics to bolster students' learning capabilities. Investigating limiting factors and supportive measures in upcoming studies would be beneficial, considering that diverse perspectives from various geographical locations and cultures globally will unquestionably enhance the research. Future studies could also provide more understanding of how to address this matter in boys' schools across varying educational contexts. While this study used questionnaires to measure students' critical thinking and problem-solving skills, future studies could assess these skills more effectively through tests, as they belong to the cognitive domain and may be better evaluated through performance-based assessments.

**Table:** Abbreviations

PBL	Project-Based Learning
PLS-SEM	Partial Least Squares-Structural Equation Model
CL	Collaborative skills
MA	Math Attitude
CTK	Critical thinking skills
PSS	Problem-Solving Skills
SE	Student Engagement

## CRediT authorship contribution statement

**Nadia Rehman:** Writing – original draft, Validation, Software, Investigation, Formal analysis, Conceptualization. **Xiao Huang:** Writing – review & editing, Supervision, Project administration, Funding acquisition, Conceptualization. **Amir Mahmood:** Writing – review & editing, Writing – original draft, Validation, Formal analysis, Data curation. **Mohammed A.M. AlGerafi:** Writing – review & editing, Validation, Resources. **Saima Javed:** Writing – review & editing, Validation.

## Informed consent

Informed consent was obtained for the present study, and all participants provided written consent. Additionally, the ethics committee reviewed and approved the need for consent.

## Availability of data and materials

The data available at https://doi.org/10.7910/DVN/MSDS9V in Harvard Dataverse can be accessed upon request.

## Ethics approval statement

This study was approved by the institutional review board and followed ethical guidelines for protecting human subjects.

## Declaration of competing interest

The authors declare that they have no known competing financial interests or personal relationships that could have appeared to influence the work reported in this paper.
