# Supplementary material for: Project-based learning as a catalyst for 21st-Century skills and student engagement in the math classroom
Source: Heliyon. 2024 Nov 15;10(23):e39988. doi: 10.1016/j.heliyon.2024.e39988 (PMC11629213; doi:10.1016/j.heliyon.2024.e39988)
Supplement: Multimedia component 1 [file mmc1.docx]

| Construct | Items |
| --- | --- |
| Project-based learning | Solving problems with a group of friends made the problem-solving process easier to manage. |
|  | I gained more confidence in solving mathematics problems through a PBL approach because of help from friends and teachers. |
|  | The challenge of solving the problem task kept me going and thinking. |
|  | I enjoyed working on math projects with my friends |
|  | PBL helped me figure out mathematical problems that do not appeal to me |
|  | PBL changed my perception of math learning |
|  | PBL enhanced the skills required for solving mathematical problems |
|  | PBL helped me to be more in the learning process |
|  | PBL helped me to develop analytical and problem-solving skills in mathematics |
|  | PBL changed my approach to mathematics |
|  | I would prefer this approach (PBL) of solving mathematics problems than solving textbook problems. |
|  | This PBL approach makes mathematics more interesting and challenging. |
|  | PBL significantly enhanced my understanding of a mathematical concept |
|  | PBL helped me to adapt to challenges or obstacles in understanding mathematical concepts |
| Collaborative Learning skills | The participants of the discussion forum provided helpful feedback to each other regarding the course project. |
|  | I kept the necessary materials and information for the project for myself |
|  | I made a unique contribution to the joint effort. |
|  | I tried to discuss concepts being learned with others. |
|  | I felt responsible for observing other participants' work. |
|  | Sometimes, I tried to give direction to the group's work. |
|  | I tried to compliment people when I liked something they had done. |
|  | We monitored each other's work to ensure the high quality of the course project. |
|  | I felt that we depended on each other while working on the course project. |
|  | From time to time, I checked for other participants' understanding of the learned Materials |
|  | The participants of the discussion forum tried to find ways to solve group problems. |
|  | The participants of the discussion forum provided helpful feedback to each other regarding the course project. |
| Math attitude | I have usually enjoyed studying mathematics in school |
|  | I like to solve new problems in mathematics |
|  | I would prefer to do an assignment in mathematics than to write an essay |
|  | I like mathematics |
|  | I am happier in a mathematics class than in any other class |
|  | Mathematics is a very interesting subject |
|  | Winning a prize in mathematics would make me feel unpleasantly conspicuous |
|  | I am comfortable expressing my ideas on how to look for solutions to a difficult problem in mathematics. |
|  | I am comfortable answering questions in mathematics class. |
|  | Mathematics is dull |
|  | When a math problem arises that I can’t immediately solve, I stick with it until I have a solution |
|  | I am challenged by math problems I can’t understand immediately |
| Critical thinking skills | Project-based learning helped me to connect mathematical concepts to real-world situations, enhancing my critical thinking. |
|  | I noticed an increase in my curiosity and interest in understanding the 'why' behind mathematical rules and formulas. |
|  | Through project-based learning, I developed a systematic approach to solving problems and making decisions based on logical reasoning. |
|  | This approach has taught me the importance of viewing mathematical problems from multiple angles before deciding on a solution. |
|  | The iterative nature of project work has improved my patience and thoroughness in addressing complex mathematical tasks. |
|  | The hands-on nature of project-based learning made me more persistent in solving mathematical challenges. |
|  | I feel more confident in my ability to analyze and break down mathematical problems because of project-based learning experiences. |
|  | Collaborating with peers on projects led me to appreciate different perspectives and strategies for tackling mathematical questions. |
|  | This approach will help me tackle unfamiliar and challenging problems in the future. |
|  | Project-based learning encouraged me to question the standard methods and explore alternative solutions in mathematics. |
|  | This approach helps me to think of multiple solutions |
|  | I found myself more actively engaged in solving complex problems during project-based learning activities. |
| Problem-Solving Skills | Project-based learning equipped me with practical problem-solving skills applicable beyond the classroom |
|  | hands-on learning in math projects made problem-solving more engaging and effective |
|  | Project-based learning allowed me to grow as a problem-solver in mathematics |
|  | Project-based learning showed me the real-life applications of math problem-solving |
|  | Project-based learning enhanced my confidence in tackling complex math problems |
|  | Working on math projects with peers helped me improve my teamwork and collaboration skills |
|  | Project-based learning sparked my interest in mathematics problem-solving |
|  | Through project-based learning, I learned to reflect on my problem-solving strategies and improve them |
|  | I discovered my creative side when solving math problems through project-based learning |
|  | I can relate project-based learning to my ability to solve real-world math problems |
| Students Engagement | Project-based learning makes me eager to explore and apply math in different contexts |
|  | Project-based learning helps me see the practical relevance of math in my daily life. |
|  | Project-based learning in math encourages me to actively participate in class. |
|  | Through project-based learning, I feel more connected to the mathematical concepts we are studying. |
|  | Project-based learning in math class sparks my curiosity and interest in the subject. |
|  | project-based learning has increased my engagement with mathematics, making it a more enjoyable experience |
